# *Leishmania infantum* recombinant kinesin degenerated derived repeat (rKDDR): A novel potential antigen for serodiagnosis of visceral leishmaniasis

**DOI:** 10.1371/journal.pone.0211719

**Published:** 2019-01-31

**Authors:** Lucas Dhom-Lemos, Agostinho Gonçalves Viana, João Luis Reis Cunha, Mariana Santos Cardoso, Tiago Antônio Oliveira Mendes, Guilherme Rafael Gomide Pinheiro, Williane Fernanda Siqueira, Francisco Pereira Lobo, Leandro Freitas Teles, Lilian Lacerda Bueno, Silvio Fernando Guimarães-Carvalho, Daniella Castanheira Bartholomeu, Ricardo Toshio Fujiwara

**Affiliations:** 1 Instituto de Ciências Biológicas, Universidade Federal de Minas Gerais, Belo Horizonte, Brazil; 2 Programa de Pós-graduação em Ciências da Saúde, Centro de Ciências Biológicas e da Saúde, Universidade Estadual de Montes Claros, Montes Claros, Brazil; Instituto Oswaldo Cruz, BRAZIL

## Abstract

Visceral leishmaniasis (VL) or kala-azar, the most severe form of leishmaniasis, can lead to death if not properly diagnosed and treated. Correct identification of infected patients and reservoirs is vital for controlling the spread of leishmaniasis. Current diagnostic kits for leishmaniasis show high sensitivity and specificity, but can also result in false negatives and cross reactions with related parasitic infections. New diagnostic methods with greater accuracy are urgently needed for diagnosis of leishmaniasis. In this study, we aimed to uncover a new highly effective antigen for the diagnosis of visceral leishmaniasis in dogs and humans, aiming to improve the accuracy compared with those of current methods of diagnosis. Initially, *in-silico* epitope prediction analyses identified several potential B-cell epitopes in the repetitive region of *Leishmania infantum* kinesin, which co-localized with predicted structural disordered regions, suggesting high potential for antigenicity. Based on this analysis, 8.5 genomic motifs, which encode the repetitive sequence of 39 degenerate amino acids, were selected for recombinant expression. BLASTn analysis of this repetitive region indicated that it is absent in the *T*. *cruzi* parasite, which is closely related to *Leishmania*, indicating the specificity of this region. This potentially antigenic protein, named recombinant kinesin degenerated derived repeat (rKDDR), was recombinantly expressed in *Escherichia coli* BL21-Star using the pET28a-TEV expression vector. We then evaluated the performance of rKDDR in correctly diagnosing *Leishmania* infection and compared this new assay with currently used diagnostic tests for leishmaniasis. rKDDR showed greater sensitivity and specificity in correctly diagnosing leishmaniasis both in human (sensitivity 92.86% and specificity 100%) and canine (sensitivity 88.54% and specificity 97.30%) sera compared with those of rK39 (human: sensitivity 90.48% and specificity 97.92%; canine: sensitivity 78.13% and specificity 90.09%). In addition, the rKDDR-ELISA outperformed the EIE-LVC kit, which is the serologic kit recommended by the Brazilian Ministry of Health for the diagnosis of canine visceral leishmaniasis. These results indicate that rKDDR is a highly promising candidate for diagnosis of visceral leishmaniasis, and is more accurate than the currently used gold-standard antigens.

## Introduction

Visceral leishmaniasis (VL), also known as Kala-azar, is a severe and highly lethal disease caused by two species of protozoan parasites, *Leishmania infantum* and *L*. *donovani*. *L*. *infantum* and *L*. *donovani* are members of the *Leishmania donovani* complex, However, recent publications have also suggested that other *Leishmania* species, such as *L*. *amazonensis*, can cause visceral leishmaniasis [1]. While *L*. *infantum* is zoonotic in Europe, North Africa, and Latin America, *L*. *donovani* is anthroponotic in East Africa and the Indian subcontinent [1]. VL is classified as a neglected tropical disease that occurs in 65 countries; 90% of the cases are concentrated in Bangladesh, India, Nepal, Sudan, and Brazil [2]. Brazil is the third most relevant endemic area in the world and presents the highest number of reported VL cases in the Americas. The number of new cases has been increasing due to the steady growth of infected dog population [3,4].

In Brazil, dogs are the main reservoirs of *L*. *infantum*. Therefore, correct identification and euthanasia of dogs harboring the parasite is one of the main targets of leishmaniasis control measures [5]. However, this aggressive practice of canine elimination has been shown ineffective because the number of new cases and mortality from VL have continued to increase. This may be due to failure to correctly identify positive animals [3,4,6].

Control of zoonotic VL relies on three main measures: proper diagnosis and treatment of human patients, euthanizing dogs that test positive via serologic diagnostic tests, and controlling the adult forms of the insect vector. Such procedures may dramatically reduce transmission [7,8] if employed long term [9]. Despite the aforementioned control efforts, the incidence of human VL is still elevated [10]. In endemic areas, over 80% of infected dogs are asymptomatic [11] and cannot be properly diagnosed by serological tests. Acquiring reliable serodiagnostic tests to efficiently detect asymptomatic infected dogs remains one of the most important issues in the control of VL.

Among the antigens used to detect VL, the rK39 antigen shows greater sensitivity and specificity for the diagnosis of VL than do rK26, rK28, and rKE16 [12]. One of the best available diagnostic tools currently available for VL diagnosis is an immunochromatographic test (ICT) based on the rK39 antigen; this test shows excellent performance (93–100% sensitivity and 97–98% specificity) in many endemic countries [13–16]. The rK39 is a series of 39-amino acid-long repeats that are part of the kinesin protein (TriTrypDB: Linj.14.1180) in *L*. *infantum* [17]. Kinesins are a superfamily of motor proteins that are present in all eukaryotes, and play important roles in regulating mitotic processes and controlling flagellar length in the *Leishmania* species [18,19]. The high antigenicity of recombinant proteins, derived from *Leishmania* kinesins, is related to long repetitive motifs in the kinesin amino acid sequence [17,20–22]. In the current study, we describe the engineering and performance of a new recombinant kinesin degenerated derived repeat, rKDDR, isolated from *L*. *infantum*. ELISA assays using rKDDR, rK39, and the EIE-LVC kit were compared using canine sera, while ELISA assays using rKDDR and rK39 were compared using human sera. Our results show that the rKDDR-ELISA showed the highest sensitivity and specificity compared with that using rK39 and that of the EIE-LVC kit. This shows that rKDDR-based assays have potential application in immunodiagnosis of VL.

## Materials and methods

### Human and dog sera

This was a retrospective study using a total of 132 human sera samples divided into three groups: 84 sera samples from patients with human with VL (HVL), 17 sera samples from patients with chronic Chagas disease (Tc) (used to evaluate cross-reactivity with *T*. *cruzi*), and 31 sera samples from healthy individuals serving as negative controls (NC). Parasitological positivity for *L*. *infantum* in the HVL group was confirmed by microscopic analysis of biopsied bone marrow aspirates and by qPCR assays specific for *Leishmania* kDNA [23]. Patient medical records were used to obtain information on results of clinical evaluation and PCR assays. These parameters were the main eligibility criteria for human samples. All sera samples were convenience series obtained from the Hospital Clemente de Farias (Montes Claros, Minas Gerais State, Brazil). Infection with *T*. *cruzi* in patients with Chagas disease was confirmed by hemoculture or by combined positivity indicated by Chagatest-ELISA Recombinante version 3.0 kit (Wiener Laboratorios, Santa Fé, Argentina) and Chagatest Indirect Hemagglutination Assay (IHA; Wiener Laboratorios).

The canine serum panel consisted of 207 samples, of which 96 samples (CVL) were from dogs naturally infected with *L*. *infantum*; these samples were obtained from an area endemic for canine VL in the Minas Gerais State, Southeast Brazil. Infection with *L*. *infantum* in dogs was confirmed by microscopic analysis of bone marrow aspirates. This was the main eligibility criterion for CVL sera samples used in this study. Sera of dogs parasitologically negative for *Leishmania*, but infected with *Babesia* (n = 15) or experimentally infected with *T*. *cruzi* (Tc, n = 15), were used to assess cross-reactivity with these parasites. Eighty-one sera samples acquired from dogs in an area non-endemic for VL, showing negative results for *Leishmania* as assessed by microscopic analysis of bone marrow aspirates, were used as the negative control (NC) group.

### Ethical statement

This study was performed in accordance with the guidelines of Brazilian College of Animal Experimentation (COBEA), following the Brazilian law for ‘‘Procedures for the Scientific Use of Animals” (11.794/2008). This study was approved by the Ethics Committee on Animal Use (protocol number 44/2012) of the Federal University of Minas Gerais (UFMG). All experiments involving human samples were approved by the Research Ethics Committee (COEP) (00842112.2.0000.5149) of the Federal University of Minas Gerais (UFMG). All human sera samples were anonymized. The Informed Consent Form (ICF) for research involving human samples was approved by COEP/UFMG. Participating individuals signed an Informed Consent Form agreeing to participate in the study. For patients younger than 18 years of age, the parents signed the Informed Consent Form, consenting to the child's participation.

### Bioinformatic prediction of B-cell linear epitopes and structural disorder

We next assessed the antigenic potential of rKDDR. For this, the complete amino-acid sequence of rKDDR was analyzed using linear prediction of B-cell epitopes via BepiPred 1.0 program with a cut-off of 1.3 [24]. We then evaluated the degree of structural disorder in the kinesin sequence using the IUPred program with a cut-off of 0.5 [25]. The antigenicity graph was generated by combining the scores obtained from the BepiPred and IUPred predictors using Perl script.

### Cloning the *KDDR* gene

To amplify the repetitive-region fragment of the kinesin gene from *L*. *infantum* genomic DNA, we designed the *KDDR* primers (forward: 5’ GCTAGCCGTGAAAGCGCCTGC 3’) and (reverse: 5’ CTCGAGTCAGGCCTCCAGCTGA 3’), containing the sites for restriction enzymes *Nhe*I (GCTAGC) and *Xho*I (CTCGAG), respectively, at the 5' ends to facilitate cloning. The PCR product was excised from the gel, purified, and cloned into a *p*GEM-T Easy Vector (Promega, USA). Competent cells from *Escherichia coli* XL1-Blue (Phoneutria, Brazil) were transformed with the recombinant plasmid *p*GEM/KDDR. After positive clones were confirmed by digestion with the enzymes *Nhe*I and *Xho*I, the KDDR insert was subcloned into a bacterial expression vector *p*ET28a-TEV. Electrocompetent *E*. *coli* BL21-Star (Thermo Fisher Scientific, USA) cells were transformed with the recombinant plasmid *p*ET28a-TEV/KDDR by electroporation using a MicroPulser Electroporation Apparatus (Bio-Rad Laboratories, USA). Correct gene insertion was confirmed by colony PCR and Sanger sequencing using T7 primers (Macrogen, South Korea).

### Expression and purification of recombinant KDDR

The expression of rKDDR in transformed *E*. *coli* cells was induced after the addition of 1mM isopropyl β-D-1-thiogalactopyranoside (IPTG) and incubation for 3 h at 37°C at 200 rpm. Cells were ruptured by sonication, and soluble fractions were obtained by centrifugation. The recombinant protein was purified using Ni^2+^ affinity chromatography with HisTrap HP 5 mL column (GE Healthcare, USA) coupled to an ÄKTA Prime Plus system (GE Healthcare, USA). The purified rKDDR protein, having 362 amino acids and predicted molecular weight of 40.2 kDa, was separated by SDS-PAGE.

### Enzyme-linked immunosorbent assay

rKDDR ELISA (SafeTest Diagnostic, Brazil), as well as rK39 ELISA and the EIE-LVC kit (FIOCRUZ-Bio-Manguinhos, Brazil), were used according to the manufacturer’s instructions. rK39 ELISA and the EIE-LVC are used as reference tests in laboratory assays available commercially for the diagnosis of visceral leishmaniasis. In this study, 96-well ELISA microplates (Costar, USA) were coated with 50 ng of recombinant antigen diluted in 100 μL carbonate buffer [15 mM Na_2_CO_3_ (Synth, Brazil); 34 mM NaHCO_3_ (Merck, Brazil); pH = 9.6] and incubated for 16 hours (overnight) at 4°C. Plates were blocked with 150 μl of 2% casein in phosphate buffered saline (PBS; pH = 7.4) for 2 hours at room temperature. After blocking, 100 μL of human or canine sera, diluted at 1:100 in a solution of PBS and 0.05% Tween 20 (pH 7.4), was added to the wells and incubated for 12–16 hours (overnight) at 4°C. Plates were washed five times with a washing solution of PBS and 0.05% Tween 20. Then, peroxidase-conjugated antibody (Sigma-Aldrich, USA), specific for human or canine IgG, was diluted 1:5000 in a solution of PBS and 0.05% Tween 20 and added to all wells at 100 μL per well. Plates were incubated at 37°C for 1 hour and 30 minutes, and then washed five times with washing solution. Finally, 100 μL of substrate solution [0.1 M citric acid, 0.2 M Na_2_PO_4_, 0.05% o-phenylenediamine dihydrochloride (OPD), and 0.1% H_2_O_2_] was added to the wells, followed by incubation at 37°C for 10 minutes. The reaction was then stopped by adding 50 μL of 4N H_2_SO_4_. Absorbance was measured in using an automated microplate ELISA reader (VersaMax, Molecular Devices, USA) at 492 nm.

### Statistical analysis

All statistical analyses were performed using GraphPad Prism software (version 5.0). We performed a sample-size calculation to determine the ideal sample number for a diagnostic accuracy study. To maximize sensitivity and specificity, the lower limit of positivity (cut-off) for each recombinant protein was determined based on Receiver Operating Characteristic (ROC) curve [26]. The performance of each assay was evaluated according to sensitivity (Se), specificity (Sp), positive predictive value (PPV), negative predictive value (NPV), area under the curve (AUC), and accuracy (AC). The degree of agreement between rKDDR ELISA, rK39 ELISA, and the EIE-LVC kit with a parasitological assay (biopsy or PCR) was determined using Kappa index (κ) values with 95% confidence intervals. Kappa index values were interpreted according to the following Fleiss scale: 0.00–0.20, poor; 0.21–0.40, fair; 0.41–0.60, moderate; 0.61–0.80, good; 0.81–0.99, very good; and 1.00, perfect [27].

## Results

### Linear B-cell epitopes and structural disorder predictions in *L*. *infantum* kinesin protein

**Fig 1:**
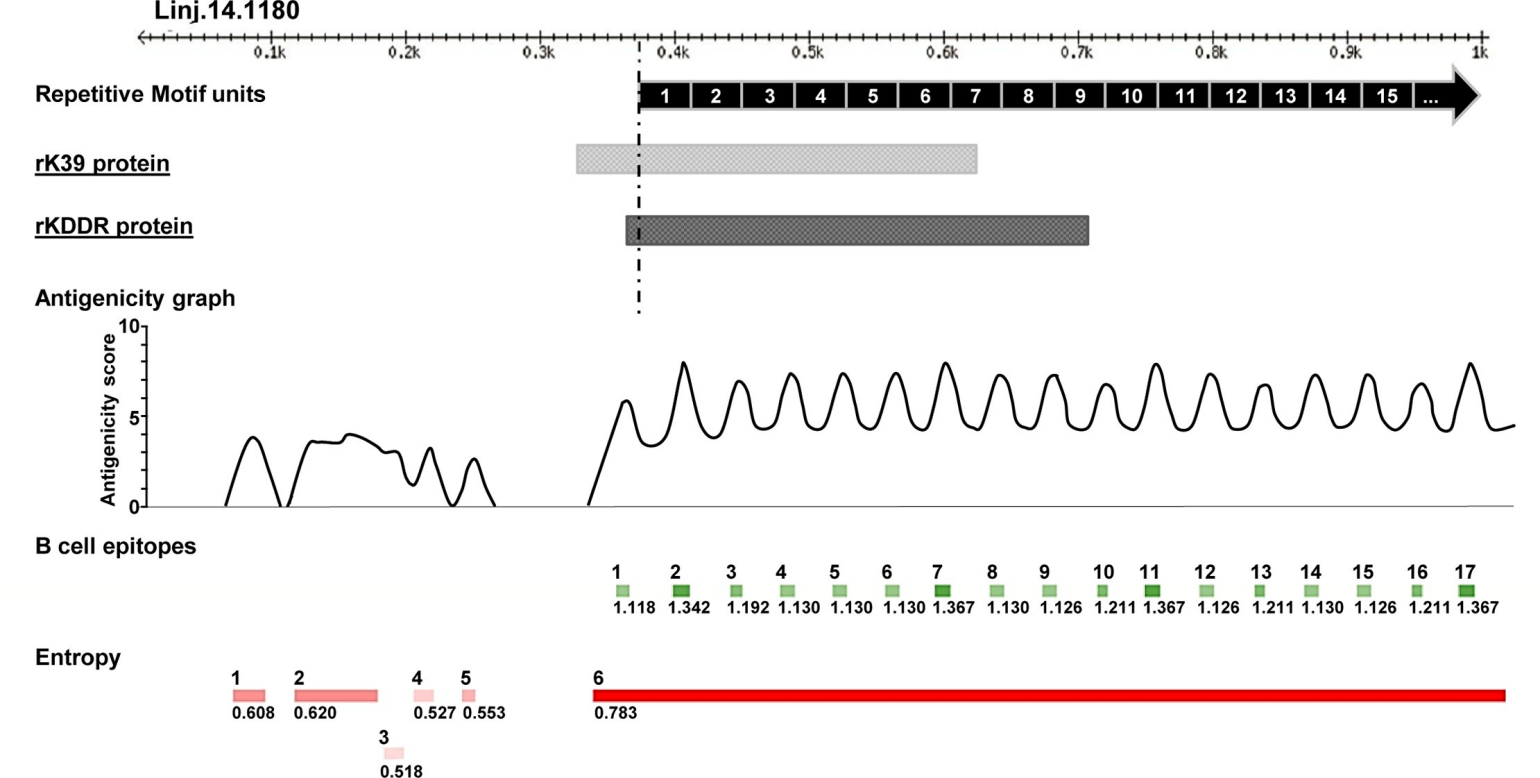
Predictions of B-cell linear epitopes and intrinsically unstructured/disordered regions (entropy) of *L*. *infantum* kinesin partial gene. The first thousand amino acids of the kinesin protein of *L*. *infantum* (TriTrypDB ID: Linj.14.1180) are represented by the black ruler at the top of the figure. Each of the numbered black boxes (1 to 15) represents the repetitive motif units of 39 amino acids of this kinesin. At the bottom of the figure, the dashed black line indicates the beginning of the repetitive sequence region. The light gray box and dark gray box represent the position and size of rK39 and rKDDR sequences, respectively. The green boxes indicate B-cell epitopes predicted by BepiPred, and the red boxes indicate disordered regions predicted by IUPred. Predicted regions as B-cell linear epitopes that are associated with a high degree of structural disorder also exhibit a high antigenicity score, as observed in the graph at the center of the figure.

In this study, we used the BepiPred 1.0 tool to predict linear B-cell epitopes in the kinesin of *L*. *infantum*. This was done to evaluate the antigenic potential of rKDDR. We also determined the degree of structural disorder along the kinesin sequence using the IUPred tool. The results of predictions, shown in Fig 1, indicate numerous predicted linear B-cell epitopes in the repetitive region of this kinesin.

Concerning the entropy observed in the kinesin, we detected a long region of structural disorder in the repeat region, as predicted by the IUPred program. This suggests a greater degree of structural linearization in this region of the kinesin. The presence of these unfolded regions in the protein likely facilitates access for antibody binding. The co-localization of disordered regions with B-cell epitope predictions reinforces the accuracy of predictions (Fig 1). Once location of the repetition blocks was also mapped on the sequence, it was possible to determine the overlap between repetitive motifs and peaks of antigenicity scores, obtained by combining the predictions described above (Fig 1).

The rK39 protein retains a substantial portion of its sequence outside the repeat region. Conversely, the rKDDR protein is mainly composed of the repetitive region (Fig 1). For this reason, rKDDR shows a greater number (9) of linear B-cell epitopes in its sequence compared with that of rK39 (7).

### Cloning, expression, and purification of soluble recombinant KDDR

**Fig 2:**
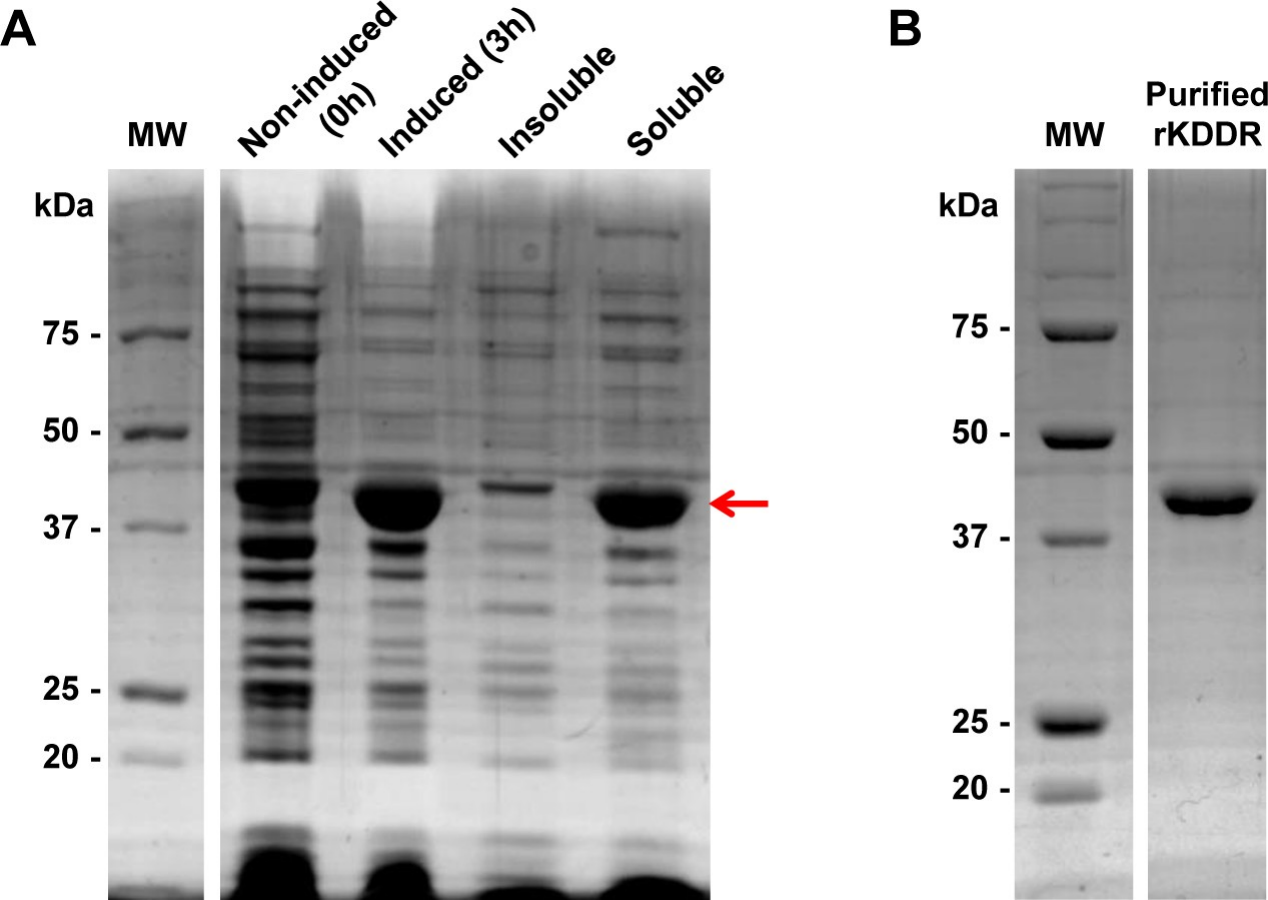
Analysis of expression in bacteria and purification of recombinant KDDR protein. **(A)** Bacterial extracts containing the plasmid *p*ET28a-TEV/KDDR were separated on 12.5% SDS-PAGE and stained with Coomassie Blue before (0 h) and after (3 h) induction with IPTG. The solubility of the protein was evaluated after bacterial lysis and separation of soluble and insoluble fractions by centrifugation. The red arrow indicates the KDDR protein band after expression. **(B)** The soluble fraction of the bacterial lysate containing rKDDR was purified by affinity chromatography, and the protein was obtained with the expected molecular weight of 40.2 kDa. MW: molecular weight; kDa: kilodalton.

The coding region of rKDDR, contained in the kinesin gene (TriTrypDB ID: Linj.14.1180) of *L*. *infantum* genomic DNA, was amplified by PCR. The PCR product was cloned into a *p*GEM-T Easy vector and subcloned into the bacterial expression vector *p*ET28a-TEV. In order to confirm the identity of the cloned KDDR, the plasmid containing the KDDR insert (*p*ET28a-TEV/KDDR) was sequenced. The construct *p*ET28a-TEV/KDDR was then transformed by electroporation into *E*. *coli* BL21-Star. The recombinant KDDR protein was expressed by IPTG induction and presented a high degree of solubility (Fig 2A). After purification by affinity chromatography, the KDDR protein with high level of purity was obtained (Fig 2B).

### Molecular analysis of rKDDR

**Fig 3:**
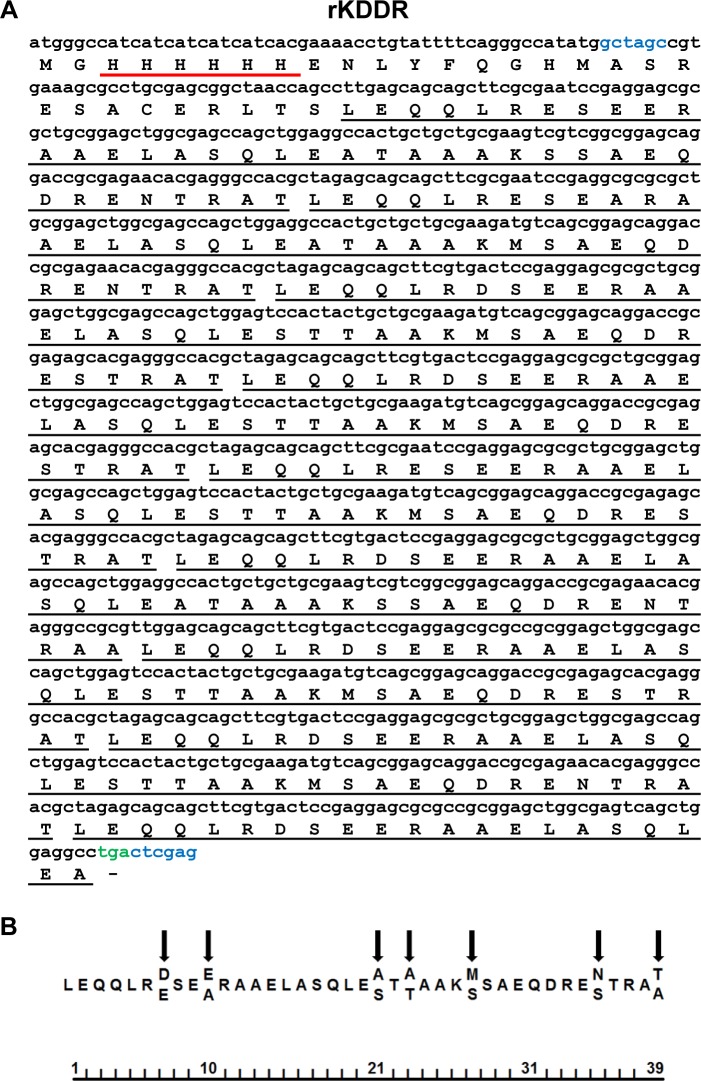
DNA sequence and predicted amino acid sequence of the KDDR protein. **(A)** Nucleotide sequence of KDDR is shown in lowercase letters, and the deduced amino acid sequence is shown in uppercase letters; underlined in black are repetitive motifs of 39 amino acids, and underlined in red are the His-Tags. Nucleotide sequences in blue correspond to sites of restriction enzymes *Nhe*I and *Xho*I, and the color green designates the stop codon sequence added. **(B)** Consensus sequence of repetitive motifs of KDDR. Arrows indicate the positions of amino-acid degeneration, and the ruler at the bottom of the figure represents the number of amino acids present in the repetitive motif.

The rKDDR protein has an open reading frame (ORF) containing 1,086 base pairs that encode 362 amino acids (Fig 3A), resulting in a predicted molecular weight of 40.2 kDa and isoelectric point (pI) of 4.63. The recombinant KDDR protein showed 8.5 blocks of 39 amino-acid repeats, with 92% of its protein sequence consisting of repetitive motifs, with remaining 8% derived from the plasmid and from a small part (10 amino acids) of the non-repetitive portion of the kinesin. There is some degree of degeneration when comparing the 39 amino acids derived from repetitive blocks, but substitutions are usually located in the same positions (Fig 3B).

### Validation and higher performance of rKDDR antigen in the diagnosis of canine VL

**Fig 4:**
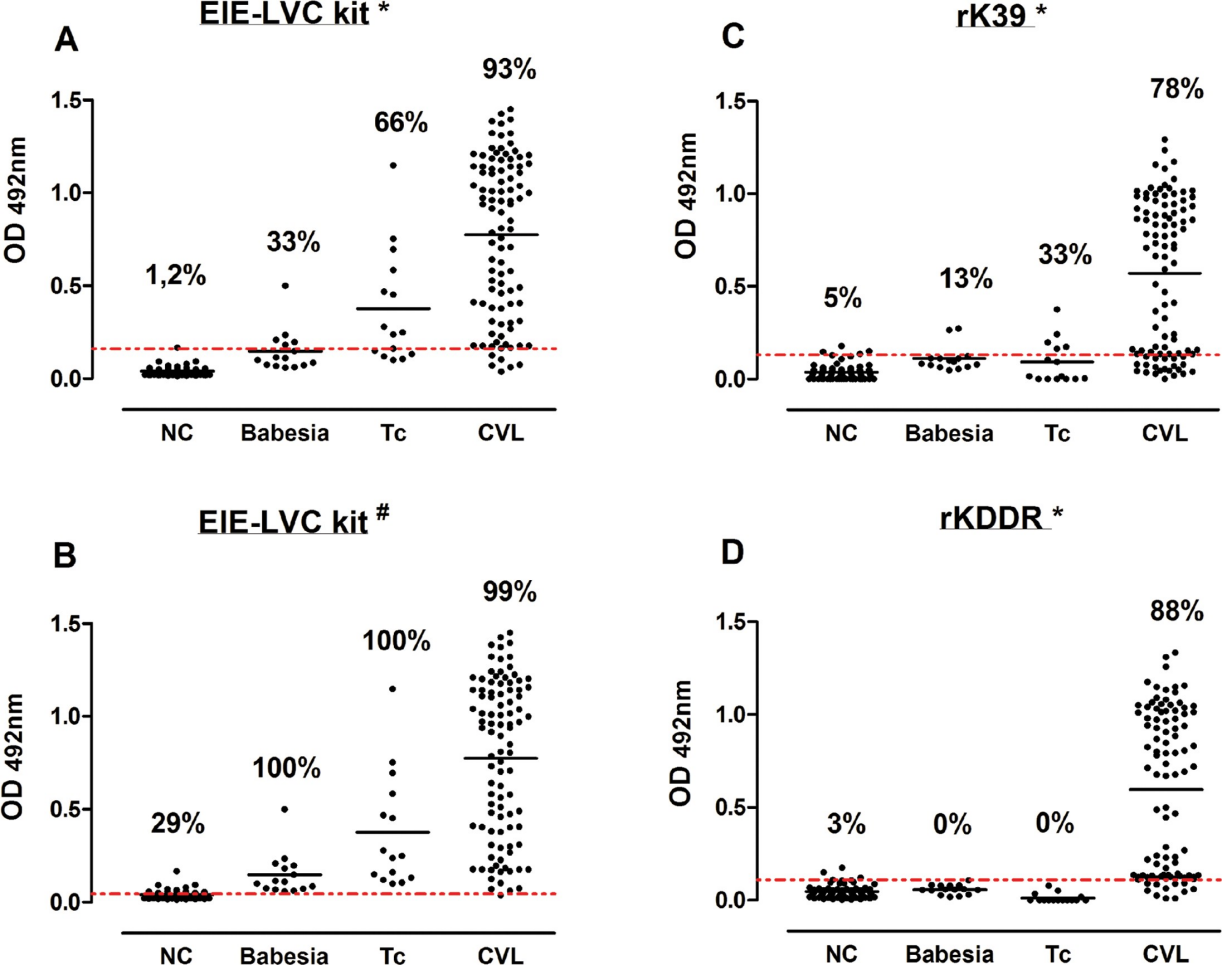
Comparing reactivity of canine sera using rK39 and rKDDR ELISA assays and the EIE-LVVLC kit. Reactivity of canine sera assessed using EIE-LVC kit **(A-B)**, analyzed with two cut-off values; rK39 **(C)**; and rKDDR **(D)**. The different ELISA assays were performed using sera from the following groups of dogs: NC, negative control (n = 81); Babesia, dogs with babesiosis to assess cross-reactivity (n = 15); Tc, dogs with Chagas disease to evaluate cross-reactivity (n = 15); and CVL, dogs with canine visceral leishmaniasis (n = 96). The y-axis shows absorbance at 492 nm. The x-axis shows different groups of canine sera. The red line shows the lower limit of positivity (cut-off). The index above each column in the plot indicates the percentage of points that are above the cut-off. The ROC curve was used to determine the cut-off for each test. *Cut-off obtained by the ROC curve; ^#^Cut-off obtained according to manufacturer’s manual.

Validation of rKDDR for serological diagnosis of CVL was performed using ELISA. The performance of rKDDR was initially compared with that of the rK39-ELISA assay because the rK39-ELISA shows excellent performance in the serodiagnosis of CVL. The performance of rKDDR was also compared with the commercially available EIE-LVC (FIOCRUZ-Bio-Manguinhos, Brazil) kit, since this kit is widely employed and recommended for diagnosis of CVL in endemic areas. We analyzed the EIE-LVC test results using two cut-off values because the cut-off values described in the manufacturer's manual are too low, implying high sensitivity and poor specificity. Our results show that the cut-off value obtained by the ROC curve (0.162) was nearly 4 times higher than the cut-off value indicated in manufacturer’s manual (0.045) (Fig 4A and 4B).

**Fig 5:**
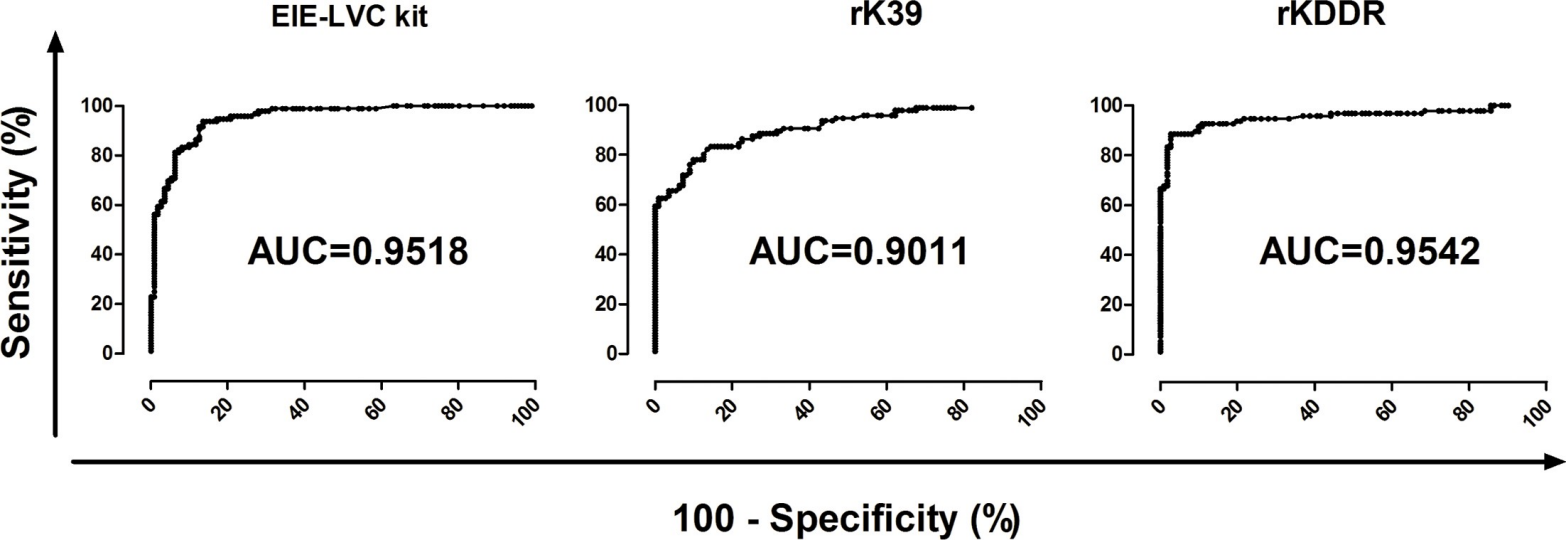
Comparison of ROC curves obtained for ELISA assays used with canine sera. ROC curves for the EIE-LVC kit, and rK39- and rKDDR-ELISA assays were generated by Prism 5.0 software; cut-offs, sensitivity, specificity, and AUC (area under the curve) were then determined. The y-axis represents the sensitivity of, and the x-axis represents the specificity of, each assay.

The profile antibody (IgG) reactivity against rKDDR presented no cross-reactivity with the *Babesia* sp. and *T*. *cruzi* (Fig 4D). Conversely, the rK39-ELISA showed lower specificity and sensitivity in diagnosing CVL diagnosis compared with those of the rKDDR ELISA (Fig 4C). The EIE-LVC kit showed higher sensitivity but lower specificity (Fig 4A and 4B) even when both cut-offs (that of the manufacturer and that obtained via ROC curve) were used. The rKDDR-ELISA showed higher sensitivity (88.54%, 95% CI: 80.42 to 94.14%) and specificity (97.30%, 95% CI: 92.30 to 99.44%) compared with those of the rK39-ELISA, which showed a sensitivity of 78.13% (95% CI: 68.53 to 85.92%) and specificity of 90.09% (95% CI: 82.96–94.95%) (Table 1). The rKDDR-ELISA showed the largest area under the ROC curve (AUC; area under curve = 0.954, 95% CI: 0.922 to 0.985) compared with that of rK39-ELISA (AUC = 0.901, 95% CI: 0.869 to 0.949) (Fig 5 and Table 2). As shown in Table 1, the rKDDR-ELISA also reached the highest positive predictive value (PPV) (96.70%), followed by that of rK39 (87.20%), and that of the EIE-LVC kit (84.90% for cut-off obtained by ROC curve; 64.18% for cut-off obtained from manufacturer's manual).

**Table 1:** Sensitivity, specificity, positive and negative predictive value, and accuracy of ELISA assays in diagnosing canine sera.

ELISA Test	*Cut-off*	Parameters^α^						
		Sensitivity (%)	95% CI	Specificity (%)	95% CI	PPV (%)	NPV (%)	AC (%)
rKDDR*	0.111	88.54	80.42–94.14	97.30	92.30–99.44	96.70	90.76	93.24
rK39*	0.131	78.13	68.53–85.92	90.09	82.96–94.95	87.20	82.64	84.54
EIE-LVC Kit*	0.162	93.75	86.89–97.67	86.49	78.69–92.23	84.90	94.05	89.37
**EIE-LVC Kit**^**#**^	0.045	98.96	94.33–99.97	52.25	42.56–61.82	64.18	98.30	73.91

Abbreviations: (CI) confidence interval; (PPV) positive predictive value; (NPV) negative predictive value; (AC) accuracy

^α^ The calculation of the parameters was performed using all dog serum samples of this work (NC, n = 81; Babesia, n = 15; Tc, n = 15; CVL, n = 96)

* Cut-off calculated based on ROC curve

^#^ Cut-off calculated according to the manual of EIE-LVC kit.

**Table 2:** Results of diagnostic agreement between rKDDR-ELISA, rK39-ELISA, and the EIE-LVC kit assessed using canine sera.

ELISA Test	AUC	95% CI	tP	tN	FP	FN	κ^α^	95% CI	Agreement^β^
rKDDR*	0.954	0.922–0.985	85	108	3	11	0.863	0.794–0.932	Very good
rK39*	0.901	0.869–0.949	75	100	11	21	0.687	0.588–0.786	Good
EIE-LVC Kit*	0.951	0.924–0.978	90	95	16	6	0.788	0.704–0.871	Good
**EIE-LVC Kit**^**#**^	NA	NA	95	58	53	1	0.494	0.393–0.595	Moderate

Abbreviations: (AUC) area under the curve; (CI) confidence interval; (tP) test positive; (tN) test negative; (FP) false positive; (FN) false negative

^α^ To calculate Kappa index, all canine serum samples was used (NC, n = 81; Babesia, n = 15; Tc, n = 15; CVL, n = 96)

^β^ Parasitological diagnostic assay was the gold standard test for calculating the Kappa index

* Cut-off was calculated based on ROC curve

^#^ Cut-off was calculated according to the manual of EIE-LVC kit.

With respect to the accuracy of the assays (Table 1), rKDDR-ELISA showed the highest accuracy (AC = 93.24%) compared with that of rK39-ELISA (AC = 84.54%) and that of EIE-LVC kit (AC = 89.37% for cut-off obtained using the ROC curve, and AC = 73.91% for cut-off obtained from the manufacturer's manual).

The degree of agreement (Table 2) between serological assays and results of parasitological assessment indicated that rKDDR-ELISA showed the best concordance index (0.863, very good), followed by that of rK39-ELISA (0.687, good), and that of the EIE-LVC kit (0.788, good, cut-off obtained via ROC curve; 0.494, moderate, cut-off indicated in manufacturer’s manual).

### Serological recognition of rKDDR in the diagnosis of human visceral leishmaniasis

**Fig 6:**
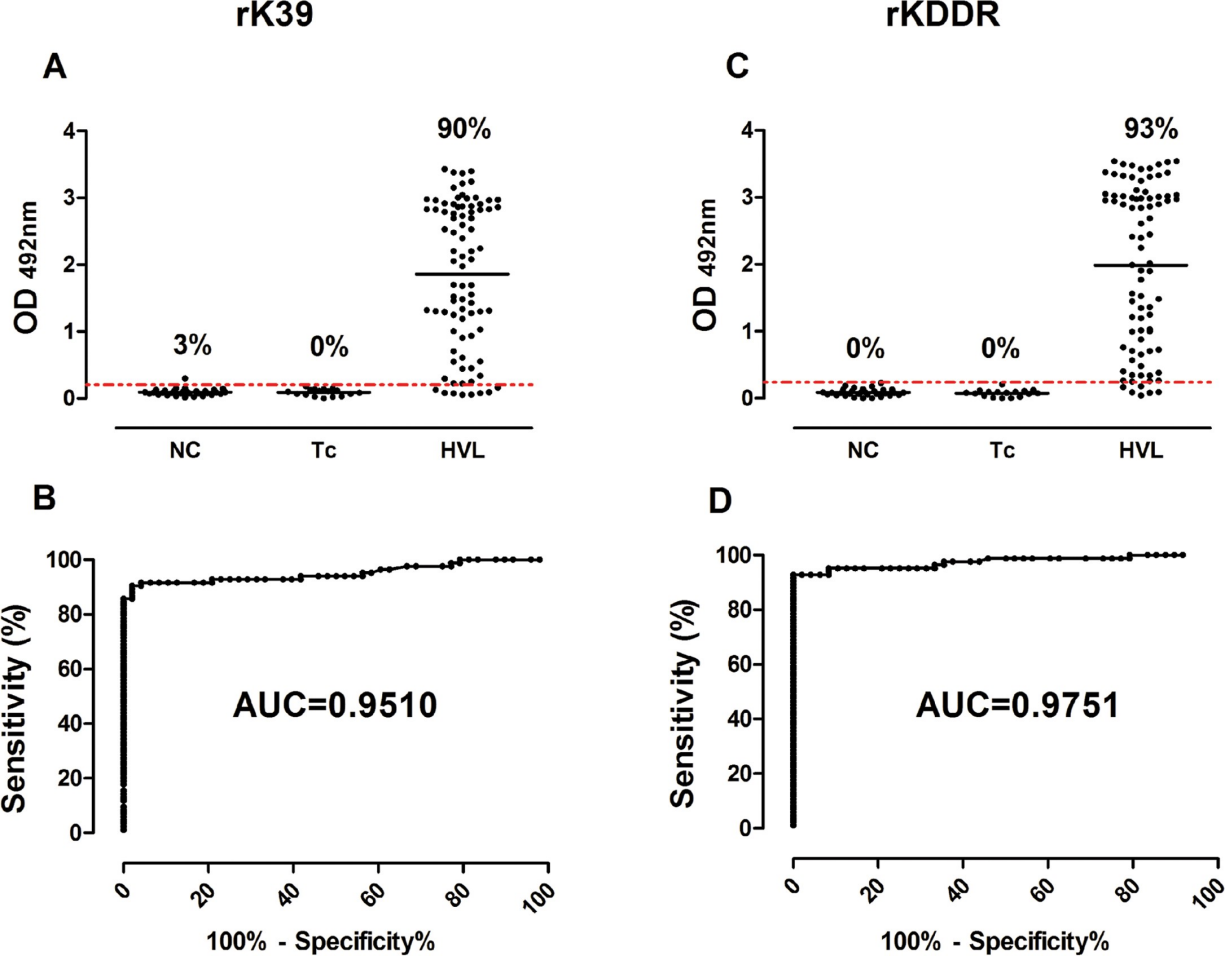
Comparison of the reactivity of human sera and ROC curves rK39 and rKDDR ELISA assays. Two ELISA assays were performed using human sera from the following groups: NC, negative control, n = 31; Tc, patients with Chagas disease to assess cross-reactivity, n = 17; HVL, patients with human visceral leishmaniasis, n = 84. In reactivity graphs, the y-axis represents absorbance at 492 nm and x-axis shows the different groups of human sera. In the ROC curves, the sensitivity of each test is represented by y-axis and specificity is represented by x-axis. In these graphs, the red line indicates the lower limit of positivity (cut-off), and the index above each column indicates the percentage of points above the cut-off. The ROC curves were generated by Prism 5.0 software and used to determine the cut-off, sensitivity, specificity, and AUC (area under the curve) for each assay.

Validation of rKDDR in serological diagnosis of HVL was performed by comparing the rKDDR and rK39 ELISA assays. Data on the reactivity and ROC curve of rKDDR and rK39 are presented in Fig 6. Tables 3 and 4 summarize the results of performance and compliance of the assays. Serological profile recognition of human VL using rKDDR was superior to that using rK39. We did not detect any cross-reactivity with sera obtained from patients with Chagas disease using both recombinant proteins.

**Table 3:** Results of sensitivity, specificity, positive and negative predictive value, and accuracy of the ELISA assays assessed using human sera.

ELISA Test	*Cut-off*	Parameters^α^						
		Sensitivity (%)	95% CI	Specificity (%)	95% CI	PPV (%)	NPV (%)	AC (%)
rKDDR*	0,242	92.86	85.10–97.33	100.00	92.60–100.00	100	88.88	95.65
rK39*	0,204	90.48	82.09–95.80	97.92	88.93–99.95	98.70	85.45	93.61

Abbreviations: (CI) confidence interval; (PPV) positive predictive value; (NPV) negative predictive value; (AC) accuracy

^α^ The calculation of the parameters was performed using all human serum samples of this work (NC, n = 31; Tc, n = 17; HVL, n = 84)

* Cut-off calculated based on ROC curve

**Table 4:** Results of diagnostic agreement between rKDDR-ELISA and rK39-ELISA assessed using human sera.

ELISA Test	AUC	95% CI	tP	tN	FP	FN	κ^α^	95% CI	Agreement^β^
rKDDR*	0.975	0.950–0.999	78	48	0	6	0.904	0.830–0.979	Very good
rK39*	0.951	0.913–0.988	76	47	1	8	0.857	0.768–0.947	Very good

Abbreviations: (AUC) area under the curve; (CI) confidence interval; (tP) test positive; (tN) test negative; (FP) false positive; (FN) false negative

^α^ To calculate Kappa index, all human serum samples was used (NC, n = 31; Tc, n = 17; HVL, n = 84)

^β^ Parasitological diagnostic assays was the gold standard test for calculating the Kappa index

* Cut-off calculated based on ROC curve

The rKDDR-ELISA showed higher sensitivity (92.86%, 95% CI: 85.10 to 97.33%) and specificity (100.00%, 95% CI: 92.60 to 100.00%) compared with that of rK39 ELISA test, which showed a sensitivity of 90.48% (95% CI: 82.09 to 95.80%) and specificity of 97.92% (95% CI: 88.93 to 99.95%). The rKDDR-ELISA also showed higher PPV (100%), NPV (88.88%), and AC (95.65%) compared with those of rK39, which showed a PPV of 98.70%, NPV of 85.45%, and AC of 93.61% (Table 3). Comparison of the two ELISA assays indicated that rKDDR-ELISA showed the largest area under the ROC curve (AUC = 0.975, 95% CI: 0.950 to 0.999), whereas rK39-ELISA showed an AUC of 0.951 with 95% CI of 0.913 to 0988 (Fig 6 and Table 4).

## Discussion

There is a pressing need for more accurate diagnostic tools for the diagnosis of leishmaniasis. However, the antigens presently used in leishmaniasis serological assays show suboptimal sensitivity and specificity. Therefore, it is necessary to produce new antigenic proteins that improve diagnostic accuracy. In this study, the recombinant KDDR protein showed good antigenic potential, presenting a large density of predicted linear B-cell epitopes in their repeating units. Validation of rKDDR by ELISA demonstrated a better performance in the serodiagnosis of human and canine VL compared to that of the globally used rK39 antigen.

With the advent of B-cell epitope prediction algorithms, it is possible to engineer known antigens in order to improve their performance in serodiagnostic assays. The increasing number of genomic assemblies from different species of parasites allows the selection of antigenic proteins or protein regions that are not conserved between species, conferring high specificity to a selected antigen [28]. In addition, large-scale production of recombinant proteins allows the obtainment of a high-purity antigen at a relatively low cost.

Several studies have shown that various repetitive proteins from parasites are often targets of the host B-cell response [17,20,29–39], which may increase the efficiency of assays used to diagnose parasitic diseases [24]. Indeed, parasites of the *L*. *donovani* complex express repetitive antigens, which elicit a large production of specific antibodies against their repetitive portions, in individuals with leishmaniasis [40]. This may occur because of the antigenic nature of repetitive proteins such as the kinesin of *L*. *infantum*. Multiple copies of repetitive motifs cause a greater exposure of repetitive antigenic portions to the host immune system. This results in a production of antibodies that recognize those repetitive portions with greater avidity [41]. rKDDR is not only a repetitive recombinant protein; it is nearly exclusively composed of repetitive motifs (92%), while rK39 contains only 60% of repetitive motifs in its structure [17]. Using bioinformatic predictions, we identified numerous linear B-cell epitopes present in the kinesin of *L*. *infantum*. These linear B-cell epitopes are present in smaller numbers in rK39 [17] compared to their numbers in rKDDR. rKDDR contains 32% more linear epitopes than does rK39, which may account for the increased sensitivity of rKDDR. In our analysis, we also observed a clear overlap of repetitive block positions with positions of predicted epitopes and peaks of antigenicity. This supports the notion that increased proportion of repetitive motifs improves the diagnostic performance of these motifs.

Different antigens, such as rK39 and rK28, have been used in serological assays with the aim of improving the diagnosis of leishmaniasis. However, variation between different diagnostic methods reinforces the need for more accurate and efficient techniques [42,43]. In our study, the performance of the commercially available EIE-LVC kit (FIOCRUZ-Bio-Manguinhos, Brazil) and rK39-ELISA were evaluated and compared with that of rKDDR-ELISA using samples of canine sera. Our results show that compared with rKDDR-ELISA, the EIE-LVC kit showed the highest sensitivity at the cost of high rate of cross reactivity with related parasites even when both cut-offs were used. The Bio-Manguinhos EIE-LVC kit is recommended by the Brazilian Ministry of Health for diagnosis of VL in dogs [44]. However, comparative serological studies have shown that the EIE-LVC kit needs to be used in conjunction with another diagnostic method to reduce false-positive results and to compensate for high potential for cross-reactivity [45,46].

With canine sera, rKDDR-ELISA showed higher sensitivity (88.54%) and specificity (97.30%) compared with those of the rK39-ELISA, which showed a sensitivity of 78.13% and specificity of 90.09%. These results indicate that serological recognition of VLC using rKDDR was superior to that using rK39 and the EIE-VLC kit

We then evaluated the performance of rKDDR- and rK39-based ELISA assays in the serological diagnosis of HVL. The rKDDR-ELISA showed higher sensitivity (92.86%) and specificity (100.00%) compared with those of the rK39-ELISA, which showed a sensitivity of 90.48% and specificity of 97.92%. Although the rK39-based assay showed good performance, the serological profile recognition of human VL by rKDDR was superior to that of rK39. As mentioned before, the greater sensitivity of rKDDR may be due to the fact that rKDDR has 32% more linear epitopes than does rK39. Unlike rK39, rKDDR is composed nearly exclusively of repetitive motifs. Conversely, the presence of a larger non-repetitive sequence in rK39, which is partially conserved in other parasites, may contribute to the lower specificity of rK39 compares with that of rKDDR.

Several studies, conducted worldwide, have evaluated the performance of an immunochromatographic test (ICT) based on the rK39 antigen. A study performed in India showed a sensitivity of 98% and specificity of 89% for rK39-ICT [47]. In a study conducted in Sudan, rK39-ICT showed a good sensitivity of 92%, but a poor specificity of 59% [48]. In another study conducted in Sudan, rK39-ICT showed a sensitivity of 81% and specificity of 97% [49]. In an earlier study, conducted in Brazil, Guimarães-Carvalho et al. (2003) showed that the sensitivity and specificity of rK39-ICT were 90% and 100%, respectively [50]. However, in more recent studies, also conducted in Brazil, rK39-ICT showed high specificity but low sensitivity [51,52]. These studies, evaluating the performance of rK39-ICT in same or different regions, highlight the variability and inconsistency in the performance of rK39. It is possible that heterogeneity among the *Leishmania* parasites in different endemic regions may cause differences in the immune responses of infected hosts [53]. Therefore, despite the good performance of rK39-ICT, the limitations of this test are low sensitivity and poor specificity in some of the regions endemic for VL [51,54]. Bhattacharyya et al. showed that genetic diversity, and a polymorphism in the sequence of rK39 occurring in East Africa and South Asia, influence the performance of rK39-based assay [55]. However, the rKDDR protein contains more repetitive sequences, suggesting that this antigen can be more widely used in the various regions.

An accurate diagnostic method should provide fundamental information regarding the epidemiological status of VL, leading to the development of more effective control measures [6]. The rKDDR antigen demonstrated better serodiagnostic performance in diagnosing both human and canine VL compared to the performance of the rK39 antigen. This indicates that the rKDDR protein can be used for a more accurate diagnosis of VL. The rapid immunochromatographic test is an efficient and low-cost method for monitoring and controlling the spread of leishmaniasis. In addition, this rapid test is simple to perform in the field and does not require trained technicians. In our next study, we aim to develop an immunochromatographic method using the rKDDR protein. This can be an efficient tool fin the diagnosis of canine and human leishmaniasis.

## Supporting information

S1 STARD Checklist (DOCX)

## References

[1] Lukeš J, Mauricio IL, Schönian G, Dujardin J-C, Soteriadou K, Dedet J-P, et al Evolutionary and geographical history of the Leishmania donovani complex with a revision of current taxonomy. Proceedings of the National Academy of Sciences. 2007;104: 9375–9380. 10.1073/pnas.0703678104 17517634 PMC1890502

[2] Desjeux P. Leishmaniasis: current situation and new perspectives. Comparative Immunology, Microbiology and Infectious Diseases. 2004;27: 305–318. 10.1016/j.cimid.2004.03.004 15225981

[3] Alvar J, Vélez ID, Bern C, Herrero M, Desjeux P, Cano J, et al Leishmaniasis Worldwide and Global Estimates of Its Incidence. PLoS ONE. 2012;7: e35671 10.1371/journal.pone.0035671 22693548 PMC3365071

[4] Maia-Elkhoury ANS, Alves WA, Sousa-Gomes ML de, Sena JM de, Luna EA. Visceral leishmaniasis in Brazil: trends and challenges. Cadernos De Saúde Pública. 2008;24: 2941–2947. 19082286 10.1590/s0102-311x2008001200024

[5] Ministério da Saúde. Manual de vigilância e controle da leishmaniose visceral. Ministério da Saúde Brasília; 2006.

[6] Ministério da Saúde. Ministério da Saúde, Secretaria de Vigilância em Saúde, Boletin Epidemiológico, 2010; 2:11–13. Ministério da Saúde; 2010.

[7] Desjeux P. Leishmaniasis: Public health aspects and control. Clinics in Dermatology. 1996;14: 417–423. 10.1016/0738-081X(96)00057-0 8889319

[8] Jeronimo SMB, Teixeira MJ, Sousa A de Q, Thielking P, Pearson RD, Evans TG. Natural History of Leishmania (Leishmania) chagasi infection in Northeastern Brazil: Long-Term Follow-Up. Clinical Infectious Diseases. 2000;30: 608–609. 10.1086/313697 10722458

[9] Magalhaes PA, Mayrink W, da Costa CA, Melo MN, Dias M, Batista SM, et al [Kala-azar in the Rio Doce, Minas Gerais area. Results of prophylactic measures]. Revista Do Instituto De Medicina Tropical De São Paulo. 1980;22: 197–202. 7209275

[10] Dantas-Torres F, Brandão-Filho SP. Geographical expansion of visceral leishmaniasis in the State of Pernambuco. Revista da Sociedade Brasileira de Medicina Tropical. 2006;39: 352–356. 10.1590/S0037-86822006000400007 17119750

[11] Dantas-Torres F, de Brito MEF, Brandão-Filho SP. Seroepidemiological survey on canine leishmaniasis among dogs from an urban area of Brazil. Veterinary Parasitology. 2006;140: 54–60. 10.1016/j.vetpar.2006.03.008 16621286

[12] Sarkari B. Immunodiagnosis of visceral leishmaniasis: Current status and challenges: A review article. Iranian Journal of Parasitology. 2018;13: 331–341. 30483323 PMC6243177

[13] Singh S, Gilman-Sachs A, Chang KP, Reed SG. Diagnostic and prognostic value of K39 recombinant antigen in Indian leishmaniasis. The Journal of Parasitology. 1995;81: 1000–1003. 8544037

[14] Qu JQ, Zhong L, Masoom-Yasinzai M, Abdur-Rab M, Aksu HS, Reed SG, et al Serodiagnosis of Asian leishmaniasis with a recombinant antigen from the repetitive domain of a Leishmania kinesin. Transactions of the Royal Society of Tropical Medicine and Hygiene. 1994;88: 543–545. 7992333 10.1016/0035-9203(94)90154-6

[15] Maalej IA, Chenik M, Louzir H, Ben Salah A, Bahloul C, Amri F, et al Comparative evaluation of ELISAs based on ten recombinant or purified Leishmania antigens for the serodiagnosis of Mediterranean visceral leishmaniasis. The American Journal of Tropical Medicine and Hygiene. 2003;68: 312–320. 12685637

[16] Zijlstra EE, Nur Y, Desjeux P, Khalil EA, El-Hassan AM, Groen J. Diagnosing visceral leishmaniasis with the recombinant K39 strip test: experience from the Sudan. Tropical medicine & international health: TM & IH. 2001;6: 108–113. 11251906 10.1046/j.1365-3156.2001.00680.x

[17] Burns JM, Shreffler WG, Benson DR, Ghalib HW, Badaro R, Reed SG. Molecular characterization of a kinesin-related antigen of Leishmania chagasi that detects specific antibody in African and American visceral leishmaniasis. Proceedings of the National Academy of Sciences. 1993;90: 775–779. 10.1073/pnas.90.2.775 PMC45748 8421715

[18] Dubessay P, Blaineau C, Bastien P, Tasse L, Van Dijk J, Crobu L, et al Cell cycle-dependent expression regulation by the proteasome pathway and characterization of the nuclear targeting signal of a Leishmania major Kin-13 kinesin. Molecular Microbiology. 2006;59: 1162–1174. 10.1111/j.1365-2958.2005.05013.x 16430691

[19] Blaineau C, Tessier M, Dubessay P, Tasse L, Crobu L, Pagès M, et al A Novel Microtubule-Depolymerizing Kinesin Involved in Length Control of a Eukaryotic Flagellum. Current Biology. 2007;17: 778–782. 10.1016/j.cub.2007.03.048 17433682

[20] Bhatia A, Daifalla NS, Jen S, Badaro R, Reed SG, Skeiky YA. Cloning, characterization and serological evaluation of K9 and K26: two related hydrophilic antigens of Leishmania chagasi. Molecular and Biochemical Parasitology. 1999;102: 249–261. 10498181 10.1016/s0166-6851(99)00098-5

[21] Abass E, Bollig N, Reinhard K, Camara B, Mansour D, Visekruna A, et al rKLO8, a Novel Leishmania donovani—Derived Recombinant Immunodominant Protein for Sensitive Detection of Visceral Leishmaniasis in Sudan. PLoS Neglected Tropical Diseases. 2013;7 10.1371/journal.pntd.0002322 23875052 PMC3715527

[22] Sivakumar R, Sharma P, Chang K-P, Singh S. Cloning, expression, and purification of a novel recombinant antigen from Leishmania donovani. Protein Expression and Purification. 2006;46: 156–165. 10.1016/j.pep.2005.07.027 16172002

[23] Mary C, Faraut F, Lascombe L, Dumon H. Quantification of Leishmania infantum DNA by a real-time PCR assay with high sensitivity. Journal of Clinical Microbiology. 2004;42: 5249–5255. 10.1128/JCM.42.11.5249-5255.2004 15528722 PMC525214

[24] Larsen JEP, Lund O, Nielsen M. Improved method for predicting linear B-cell epitopes. Immunome Research. 2006;2: 2 10.1186/1745-7580-2-2 16635264 PMC1479323

[25] Dosztányi Z, Csizmok V, Tompa P, Simon I. IUPred: web server for the prediction of intrinsically unstructured regions of proteins based on estimated energy content. Bioinformatics (Oxford, England). 2005;21: 3433–3434. 10.1093/bioinformatics/bti541 15955779

[26] Linnet K, Bossuyt PMM, Moons KGM, Reitsma JBR. Quantifying the accuracy of a diagnostic test or marker. Clinical Chemistry. 2012;58: 1292–1301. 10.1373/clinchem.2012.182543 22829313

[27] Fleiss JL, Spitzer RL, Endicott J, Cohen J. Quantification of agreement in multiple psychiatric diagnosis. Archives of General Psychiatry. 1972;26: 168–171. 4551259 10.1001/archpsyc.1972.01750200072015

[28] Fernández-Robledo JA, Vasta GR. Production of recombinant proteins from protozoan parasites. Trends in Parasitology. 2010;26: 244–254. 10.1016/j.pt.2010.02.004 20189877 PMC2862126

[29] Coppel RL, Cowman AF, Anders RF, Bianco AE, Saint RB, Lingelbach KR, et al Immune sera recognize on erythrocytes Plasmodium falciparum antigen composed of repeated amino acid sequences. Nature. 1984;310: 789–792. 6382025 10.1038/310789a0

[30] Koenen M, Scherf A, Mercereau O, Langsley G, Sibilli L, Dubois P, et al Human antisera detect a Plasmodium falciparum genomic clone encoding a nonapeptide repeat. Nature. 1984;311: 382–385. 6090935 10.1038/311382a0

[31] Cowman AF, Saint RB, Coppel RL, Brown G V., Andere RR, Kemp DJ. Conserved sequences flank variable tandem repeats in two α-antigen genes of Plasmodium falciparum. Cell. 1985;40: 775–783. 10.1016/0092-8674(85)90337-X 3886159

[32] Kemp DJ, Coppel RL, Anders RF. Repetitive Proteins and Genes of Malaria. Annual Review of Microbiology. 1987;41: 181–181. 10.1146/annurev.mi.41.100187.001145 3318667

[33] Ibañez CF, Affranchino JL, Macina RA, Reyes MB, Leguizamon S, Camargo ME, et al Multiple Trypanosoma cruzi antigens containing tandemly repeated amino acid sequence motifs. Molecular and Biochemical Parasitology. 1988;30: 27–33. 3135494 10.1016/0166-6851(88)90129-6

[34] Burns JM, Shreffler WG, Rosman DE, Sleath PR, March CJ, Reed SG. Identification and synthesis of a major conserved antigenic epitope of Trypanosoma cruzi. Proceedings of the National Academy of Sciences. 1992;89: 1239–1243. 10.1073/pnas.89.4.1239 PMC48424 1371355

[35] Goto Y, Coler RN, Guderian J, Mohamath R, Reed SG. Cloning, Characterization, and Serodiagnostic Evaluation of Leishmania infantum Tandem Repeat Proteins. Infection and Immunity. 2006;74: 3939–3945. 10.1128/IAI.00101-06 16790767 PMC1489730

[36] Goto Y, Coler RN, Reed SG. Bioinformatic Identification of Tandem Repeat Antigens of the Leishmania donovani Complex. Infection and Immunity. 2007;75: 846–851. 10.1128/IAI.01205-06 17088350 PMC1828517

[37] Goto Y, Carter D, Guderian J, Inoue N, Kawazu S-I, Reed SG. Upregulated Expression of B-Cell Antigen Family Tandem Repeat Proteins by Leishmania Amastigotes. Infection and Immunity. 2010;78: 2138–2145. 10.1128/IAI.01102-09 20160013 PMC2863543

[38] Thuy NT, Goto Y, Lun Z-R, Kawazu S-I, Inoue N. Tandem repeat protein as potential diagnostic antigen for Trypanosoma evansi infection. Parasitology Research. 2012;110: 733–739. 10.1007/s00436-011-2632-9 21927872

[39] Nguyen T-T, Zhou M, Ruttayaporn N, Nguyen QD, Nguyen VK, Goto Y, et al Diagnostic value of the recombinant tandem repeat antigen TeGM6-4r for surra in water buffaloes. Veterinary Parasitology. 2014;201: 18–23. 10.1016/j.vetpar.2014.01.009 24524896

[40] Goto Y, Omachi S, Sanjoba C, Matsumoto Y. Elevation of Serum B Cell-Activating Factor Levels During Visceral Leishmaniasis. The American Journal of Tropical Medicine and Hygiene. 2014; 10.4269/ajtmh.14-0260 25157121 PMC4228886

[41] Valiente-Gabioud AA, Veaute C, Perrig M, Galan-Romano FS, Sferco SJ, Marcipar IS. Effect of repetitiveness on the immunogenicity and antigenicity of Trypanosoma cruzi FRA protein. Experimental Parasitology. 2011;127: 672–679. 10.1016/j.exppara.2010.11.011 21118687

[42] Venturin GL, Bragato JP, Silva KLO, de Lima VMF. Recombinant K28 antigen in ELISA in the diagnosis of canine visceral leishmaniosis. Parasite Immunology. 2015;37: 670–673. 10.1111/pim.12281 26408410

[43] Scalone A, De Luna R, Oliva G, Baldi L, Satta G, Vesco G, et al Evaluation of the Leishmania recombinant K39 antigen as a diagnostic marker for canine leishmaniasis and validation of a standardized enzyme-linked immunosorbent assay. Veterinary Parasitology. 2002;104: 275–285. 10.1016/S0304-4017(01)00643-4 11836028

[44] Alves WA, Bevilacqua PD. Reflexões sobre a qualidade do diagnóstico da leishmaniose visceral canina em inquéritos epidemiológicos: o caso da epidemia de Belo Horizonte, Minas Gerais, Brasil, 1993–1997. Cadernos de Saúde Pública. 2004;20: 259–265. 10.1590/S0102-311X2004000100043 15029328

[45] Menezes-Souza D, Mendes TA de O, Nagem RAP, Santos TT de O, Silva ALT, Santoro MM, et al Mapping B-cell epitopes for the peroxidoxin of Leishmania (Viannia) braziliensis and its potential for the clinical diagnosis of tegumentary and visceral leishmaniasis. PloS One. 2014;9: e99216 10.1371/journal.pone.0099216 24921246 PMC4055673

[46] Lira RA, Cavalcanti MP, Nakazawa M, Ferreira AGP, Silva ED, Abath FGC, et al Canine visceral leishmaniosis: A comparative analysis of the EIE-leishmaniose-visceral-canina-Bio-Manguinhos and the IFI-leishmaniose- visceral-canina-Bio-Manguinhos kits. Veterinary Parasitology. 2006;137: 11–16. 10.1016/j.vetpar.2005.12.020 16446034

[47] Singh DP, Goyal RK, Singh RK, Sundar S, Mohapatra TM. In search of an ideal test for diagnosis and prognosis of Kala-azar. Journal of Health, Population and Nutrition. 2010;28: 281–285. 10.3329/jhpn.v28i3.5557 PMC2980893 20635639

[48] Veeken H, Ritmeijer K, Seaman J, Davidson R. Comparison of an rK39 dipstick rapid test with direct agglutination test and splenic aspiration for the diagnosis of kala-azar in Sudan. Tropical Medicine and International Health. 2003;8: 164–167. 10.1046/j.1365-3156.2003.00996.x 12581443

[49] Ritmeijer K, Melaku Y, Mueller M, Kipngetich S, O’Keeffe C, Davidson RN. Evaluation of a new recombiant K39 rapid diagnosctic test for sudanese visceral leishmaniasis. American Journal of Tropical Medicine and Hygiene. 2006;74: 76–80. 16407349

[50] Guimarães Carvalho SF, Moreira Lemos E, Corey R, Dietze R. Performance of recombinant K39 antigen in the diagnosis of Brazilian visceral leishmaniasis. American Journal of Tropical Medicine and Hygiene. 2003;68: 321–324. 12685638

[51] Cunningham J, Hasker E, Das P, El Safi S, Goto H, Mondal D, et al A global comparative evaluation of commercial immunochromatographic rapid diagnostic tests for visceral leishmaniasis. Clinical Infectious Diseases. 2012;55: 1312–1319. 10.1093/cid/cis716 22942208 PMC3478143

[52] Moura AS, Lopes HMR de O, Mourao MVA, Morais MHF. Performance of a rapid diagnostic test for the detection of visceral leishmaniasis in a large urban setting. Revista da Sociedade Brasileira de Medicina Tropical. 2013;46: 589–593. 10.1590/0037-8682-0145-2013 24270249

[53] Abass E, Kang C, Martinkovic F, Semião-Santos SJ, Sundar S, Walden P, et al Heterogeneity of Leishmania donovani parasites complicates diagnosis of visceral leishmaniasis: Comparison of different serological tests in three endemic regions. PLoS ONE. 2015;10: 1–13. 10.1371/journal.pone.0116408 25734336 PMC4348478

[54] Kiros YK, Regassa BF. The role of rk39 serologic test in the diagnosis of visceral leishmaniasis in a Tertiary Hospital, Northern Ethiopia. BMC Research Notes. BioMed Central; 2017;10: 1–5. 10.1186/s13104-016-2345-3 28446246 PMC5407002

[55] Bhattacharyya T, Boelaert M, Miles MA. Comparison of Visceral Leishmaniasis Diagnostic Antigens in African and Asian Leishmania donovani Reveals Extensive Diversity and Region-specific Polymorphisms. PLoS Neglected Tropical Diseases. 2013;7 10.1371/journal.pntd.0002057 23469296 PMC3585016

